# Supplementary material for: p190RhoGAP proteins contain pseudoGTPase domains
Source: Nat Commun. 2017 Sep 11;8:506. doi: 10.1038/s41467-017-00483-x (PMC5593906; doi:10.1038/s41467-017-00483-x)
Supplement: Supplementary file 1 — Supplementary Information [file 41467_2017_483_MOESM1_ESM.pdf]

Description of Supplementary Files

File name: Supplementary Information
Description: Supplementary figures, supplementary tables and supplementary references.

File name: Peer review file

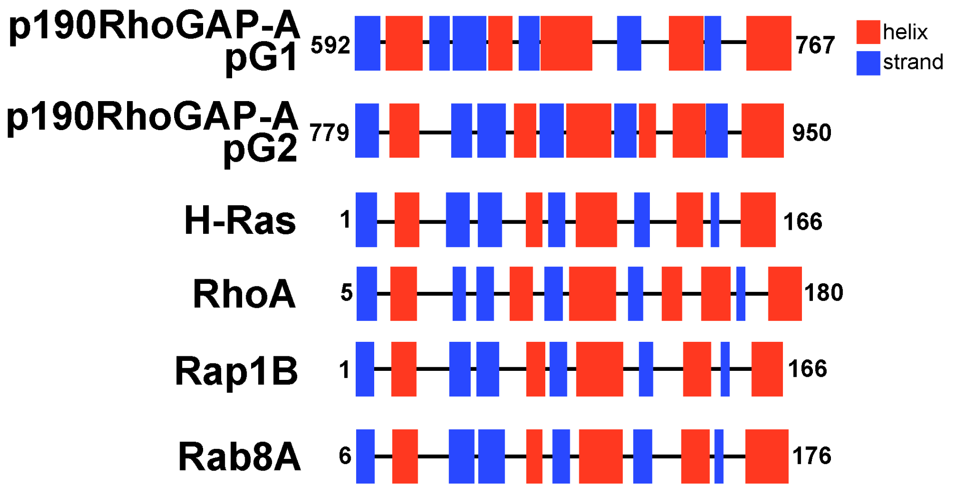

Supplementary Figure 1. Secondary structure prediction of two GTPase-like domains in the p190RhoGAP 'middle domain'.

Predicted secondary structure (HHpred) of two predicted domains within the p190RhoGAP-A (human) 'middle domain' (pG1 and pG2) compared to the assigned secondary structure of H-Ras (PDB ID: 5P21), RhoA (PDB ID: 1FTN), Rap1B (PDB ID: 3X1X), Rab8A (PDB ID: 4LHW), (determined in DSSP[1]).

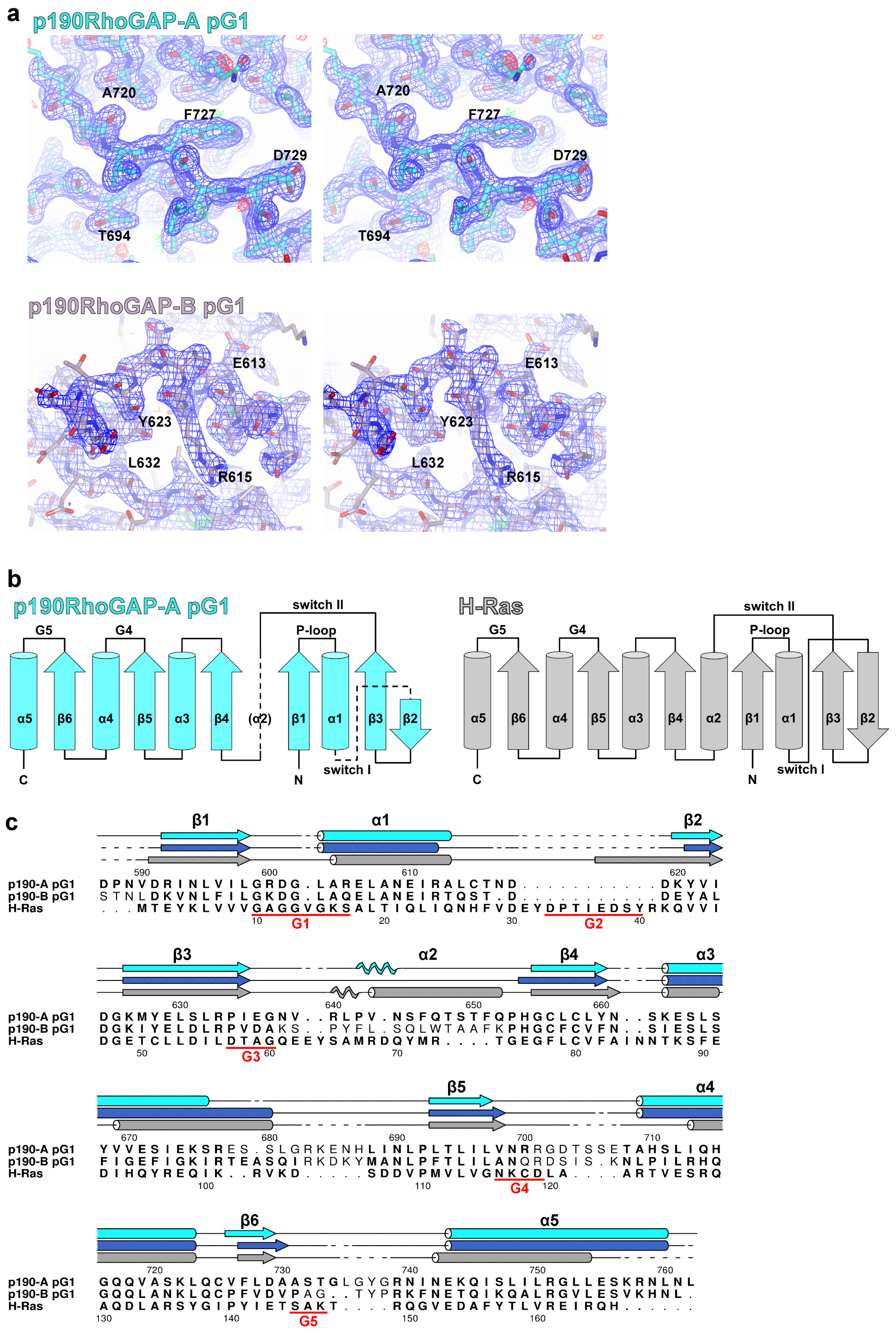

Supplementary Figure 2. Structure analysis of the p190RhoGAP pG1.

a) Representative stereoview electron density maps for p190RhoGAP-A pG1 (top) and p190RhoGAP-B pG1 (bottom). Blue: $2F_{obs}$-F_{calc} at 1σ; Green: F_{obs}-F_{calc} at +3σ; Red: F_{obs}-F_{calc} at -3σ. Some residue numbers are indicated. **b)** Topology map of p190RhoGAP pG1 (left)

compared to H-Ras (right; PDB ID: 5P21). The overall topology is similar but some differences exist, particularly in the α2, β2 and Switch I elements. Figure generated using PowerPoint (Microsoft). **c)**. Structure-based sequence alignment (DALI server) and secondary structure assignments (DSSP) for frog p190RhoGAP-A pG1, human p190RhoGAP-B pG1, and human H-Ras (PDB ID: 5P21). Residues modeled in the structure are in bold. Residue numbering for *Xenopus laevis* p190RhoGAP-A is depicted above and for H-Ras below. The figure was created using ALINE [2].

a

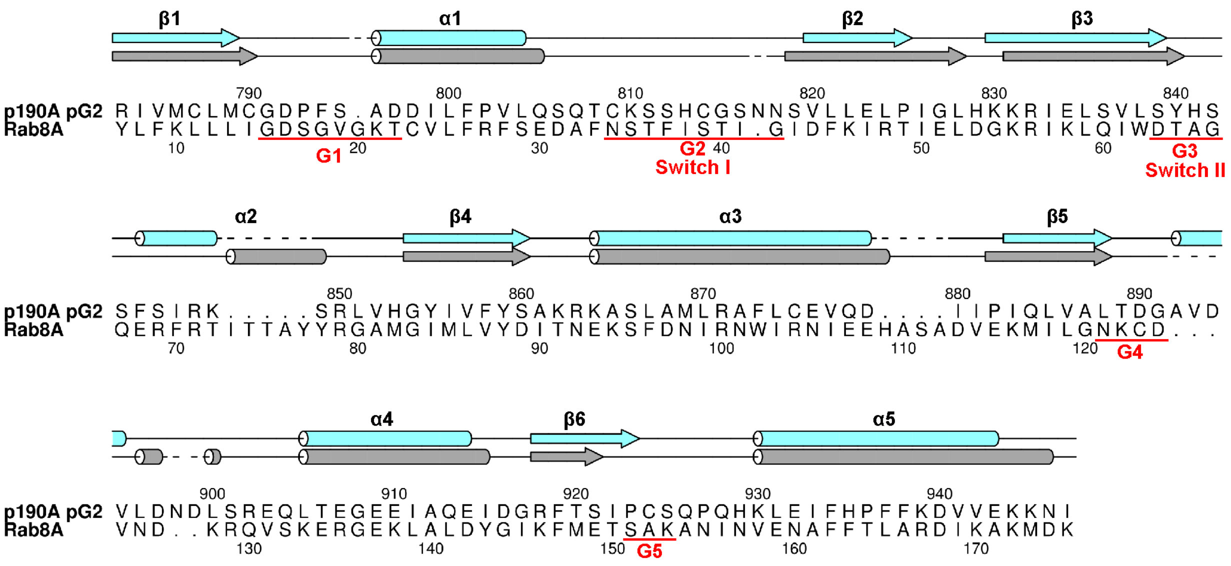

b

	P-loop G1	Switch I G2	Switch II G3	G4	G5
p190-A pG2	GDPFS.AD	CKSSHCGSNNSVL	SYHSS	LTDG	PCS
p190-B pG2	GDPFS.VD	CSAAQAGQNNSLM	SYHSS	VTDS	YSL
Rab8A	GDSGV**GKT**	FNSTFIS**T**IGIDF	**D**TA**GQ**	**NKCD**	**SAK**
Rab28A	GDGAS**GKT**	FGKQYKQ**T**IGLDF	**D**IG**GQ**	**NKID**	**SAK**
Rab26	GDSGV**GKT**	FLAGFIS**T**VGIDF	**D**TA**GQ**	**NKVD**	**SAK**
Rab21	GEGCV**GKT**	FNDKHIT**T**LGASF	**D**TA**GQ**	**NKID**	**SAK**
Rab5B	GDVGA**GKS**	FVEFQES**T**IGAAF	**D**TA**GQ**	**NKSD**	**SAK**
H-Ras	GAGGV**GKS**	FVDEYDP**T**IEDSY	**D**TA**GQ**	**NKCD**	**SAK**
Consensus	Gxxxx**GKS**	.......T.....	**D**xx**GQ**	**NK**x**D**	**SAK**

Supplementary Figure 3. Homology-based sequence alignment of p190RhoGAP pG2. a) Aligned secondary structure prediction (HHpred) of human p190RhoGAP-A pG2 and secondary structure assignment for the top HHpred hit Rab8A (DSSP; PDB ID: 4LHW, see Supplementary Table 1), are shown above the sequences. Residue numbering is included. G-motifs for Rab8A are underlined and labeled in red. **b**) Aligned G motifs from top HHpred homologous domains (Table S3) to pG2 from p190RhoGAP-A (human) showing that the predicted G motifs in pG2 are likely degraded.

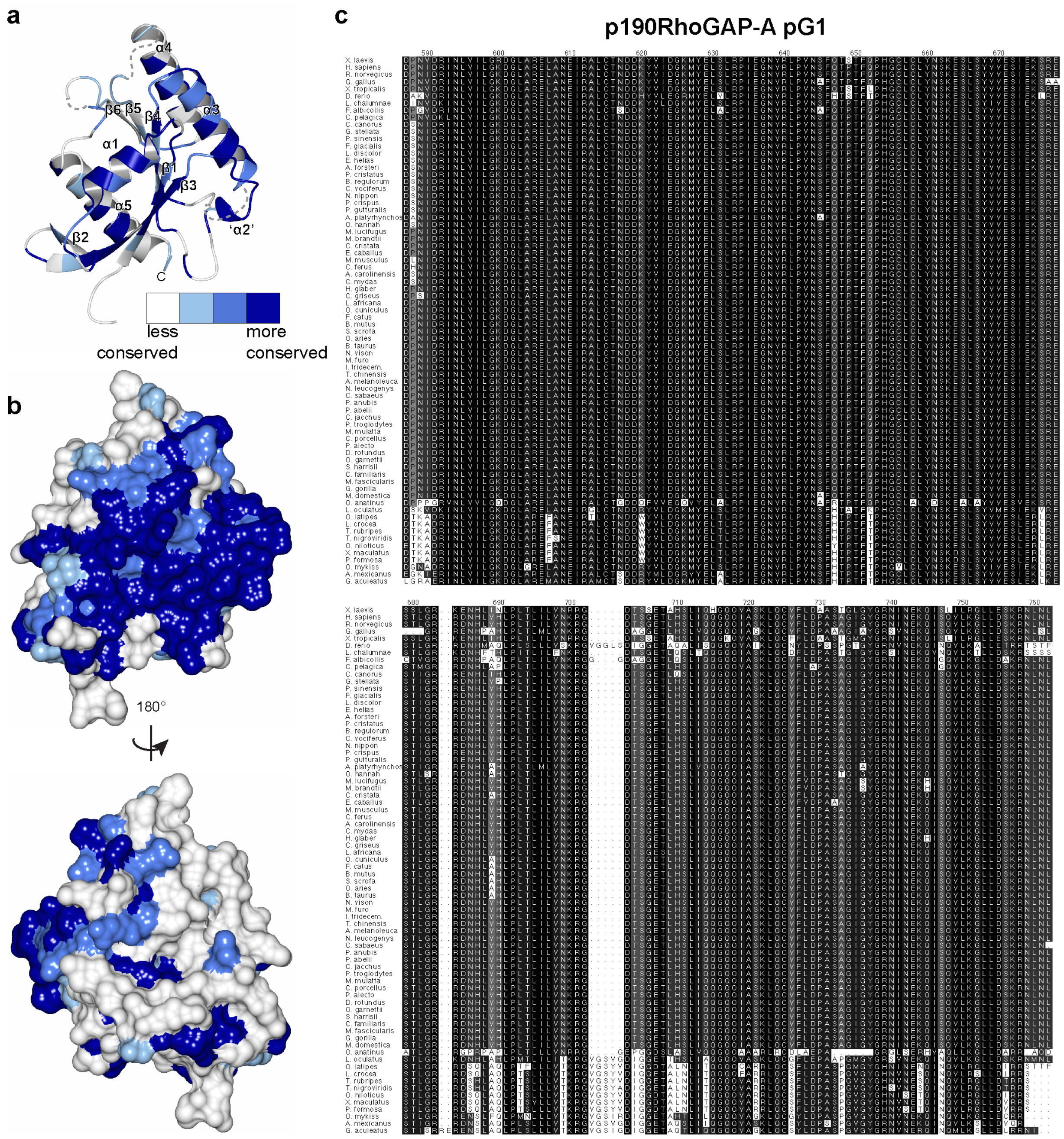

Supplementary Figure 4. Conservation analysis of p190RhoGAP-A pG1. a) Ribbon diagram of p190RhoGAP-A pG1 with residues colored according to conservation scores from ConSurf analysis[3] of the alignment in c. Dark blue indicates high conservation, white indicates low or no conservation. **b)** Surface representation of p190RhoGAP-A pG1 colored by sequence conservation. **c**) A manually curated alignment of p190RhoGAP-A pG1 regions from 74 species.

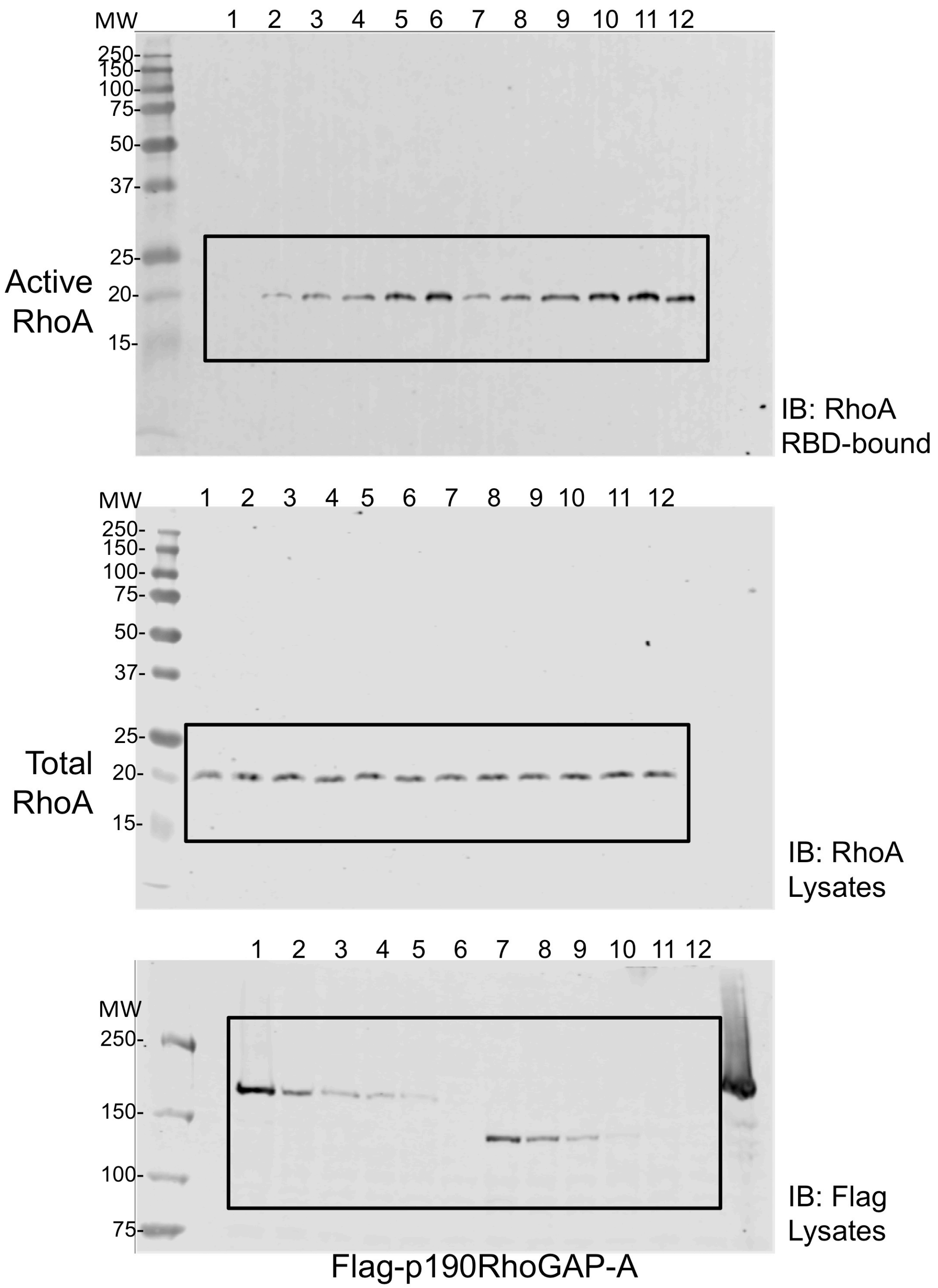

Supplementary Figure 5. Uncropped immunoblots from Figure 5 of the main text. The molecular weight markers (Bio-Rad Precision Plus All Blue), which are visible in the 700 nm channel on the LI-COR Odyssey CLx Imaging system, are included in the images and labelled.

Supplementary Table 1. Top HHpred predicted homologous domains.

	pG1						pG2					
	Human p190RhoGAP-A pG1 (residues 592-767)						Human p190RhoGAP-A pG2 (residues 779-950)					
	Protein	PDB ID	Probability (%)	E-value	Identity (%)	Number of Residues	Protein	PDB ID	Probability (%)	E-value	Identity (%)	Number of Residues
1	Rab8A	4LHW	99.9	2.5E-26	14	153	Rab8A	4LHW	99.9	1.1E-19	16	156
2	Rheb	3T5G	99.9	3.6E-25	17	157	Rab28A	2HXS	99.8	2.6E-19	13	158
3	Rap1B	3X1X	99.9	2.8E-25	15	149	Rab26	2G6B	99.8	4.3E-19	14	158
4	Rab5B	2EFE	99.9	6.7E-25	9	151	Rab21	1Z08	99.8	8.3E-19	15	152
5	Ras3	4KU4	99.9	4.3E-25	14	157	Rab5B	2EFE	99.8	3.0E-19	13	158
6	Rab5A	1R2Q	99.9	5.0E-25	12	150	Rad	3Q72	99.8	8.3E-19	13	152
7	Rab23	1Z2A	99.9	2.1E-25	14	152	Rab23	1Z2A	99.8	6.9E-19	17	156
8	Rit1	4KLZ	99.9	1.3E-25	17	156	Rit1	4KLZ	99.8	5.1E-19	16	159
9	Rab28A	2HXS	99.9	1.6E-24	9	150	Ras3	4KU4	99.8	1.4E-18	14	159
10	RalA	1U8Z	99.9	7.9E-25	14	153	Rab5A	1R2Q	99.8	1.1E-18	16	150
	Human p190RhoGAP-B pG1 (residues 590-760)						Human p190RhoGAP-B pG2 (residues 774-950)					
	Protein	PDB ID	Probability (%)	E-value	Identity (%)	Number of Residues	Protein	PDB ID	Probability (%)	E-value	Identity (%)	Number of Residues
1	Rheb	3SEA	99.5	5.8E-16	18	152	RheB	3SEA	99.2	7.5E-12	17	157
2	Rab	5UB8	99.5	3.1E-16	12	176	Centg	2IWR	99.2	1.4E-11	7	164
3	M-Ras	3KKQ	99.4	1.8E-14	17	167	Rab	5UB8	99.1	2.7E-11	14	171
4	Rap1b	3X1X	99.4	4.2E-14	15	151	Ral-B	2KWI	99.1	4.2E-11	16	163
5	Ras3	4KU4	99.4	5.3E-14	16	174	Ras-3	4KU4	99.1	2.5E-11	12	171
6	RhebL1	3OES	99.4	9E-14	17	160	Rasl12	3C5C	99.0	3.9E-11	10	160
7	Rem	3Q85	99.4	4E-14	15	153	RhebL1	3OES	99.0	7E-11	13	170
8	Rheb	3T5G	99.4	4.9E-14	17	165	Rasl12	3T5G	99.0	1.5E-10	13	163
9	Rab2B	2A5J	99.4	3.8E-14	14	159	EhRho1	3REG	99.0	3.5E-11	9	165
10	Di-Ras1	2GF0	99.4	5.8E-15	15	177	Rho6	2REX	98.9	5.7E-11	12	173
	Drosophila p190RhoGAP pG1 (residues 593-753)						Drosophila p190RhoGAP pG2 (residues 765-927)					
	Protein	PDB ID	Probability (%)	E-value	Identity (%)	Number of Residues	Protein	PDB ID	Probability (%)	E-value	Identity (%)	Number of Residues
1	Rheb	3SEA	99.5	5.8E-16	18	152	Rab8A	4LHW	99.9	1.1E-19	16	156
2	Rab	5UB8	99.5	3.1E-16	12	176	Rab28A	2HXS	99.8	2.6E-19	13	158
3	M-Ras	3KKQ	99.4	1.8E-14	17	167	Rab26	2G6B	99.8	4.3E-19	14	158
	Sponge p190RhoGAP pG1 (residues 610-774)						Sponge p190RhoGAP pG2 (residues 796-950)					
	Protein	PDB ID	Probability (%)	E-value	Identity (%)	Number of Residues	Protein	PDB ID	Probability (%)	E-value	Identity (%)	Number of Residues
1	Rab8A	4LHW	100	2.2E-30	13	154	Rab8A	4LHW	99.9	1.6E-21	16	151
2	Rap1B	3X1X	100	2.6E-29	13	153	Rab23	1Z2A	99.9	9.3E-21	14	147
3	RalA	1U8Z	100	7.2E-29	14	154	R-Ras	2FN4	99.9	1.2E-20	16	143

Uniprot IDs for Human (*Homo sapiens*) p190RhoGAP-A, Q9NRY4, and p190RhoGAP-B, Q13017. Uniprot IDs for Drosophila (*Drosophila melanogaster*), Q9VX32. NCBI Reference sequence for Sponge (*Amphimedon queenslandica*), XP_003385690.2.

Supplementary Table 2. Structure-based comparison of conserved G-motifs for selected small GTPases and pseudoGTPases.

	P-loop	Switch I	Switch II		
	G1	G2	G3	G4	G5
Consensus	GxGxxGKS	.......T.....	DxxGQ	NKxD	SAK
H-Ras	GAGGVGKS	FVDEYDPTIEDSY	DTAGQ	NKCD	SAK
Rad (RGK)	GAPGVGKS	P..EAEAAG.HTY	DIWEQ	NKSD	SAA
Rnd3 (Rnd)	GDSQCGKT	FPENYVPTVFENY	DTSGS	CKSD	SAL
AGAP2	GDARSGKS	YQV-LEKTESEQY	EEAGA	TQDR	CAT
p190-A pG1	GRDG.LAR	(deletion)	PIEGN	VNRR	AST
p190-B pG1	GKDG.LAQ	(deletion)	PVDAK	ANQR	PAG
LIC	GGTVDSQR	RR(+10)FALGYT	YTLTD	QNAE	TPS
CENP-M	GTEDALLQ	(deletion)	LAKSL	TGAG	DLE

Red indicates residues that do not match the consensus.

Supplementary Table 3. Synthetic DNA sequences.

p190RhoGAP-A pG1 (*Gallus gallus*)	GGATCCGACCCGAACGTGGACCGTATCAACCTGGTTATTCTGG<br>GCAAAGATGGCCTGGCTCGTGAACTGGCAAATGAAATCCGTGC<br>TCTGTGTACCAACGATGACAAATATGTCATTGAAGGCAAAATG<br>TACGAACTGTCCCTGCGTCCGATCGAGGGTAACGTCCGCCTGC<br>CGGTGAATGCCTTTCAGACCCCGACGTTCCAACCGCATGGCTG<br>CCTGTGTCTGTATAATAGCAAAGAAAGCCTGTCTTACGTGGTT<br>GAAAGTATTGAAAAATCCGCCGCGGGTCGTCGCGAAAACCATC<br>CGGCACACCTGCCGCTGACCCTGATGCTGGTTAATAAACGTGG<br>TGATGCAGGCGGTGAAACGCTGCACAGTCTGGTGCAGCAAGGC<br>CAGCAAATCGCTGGTAAACTGCAGTGCGTTTTTCTGGACCCGG<br>CAGCTGCGGGCATTGCATATGGTCGTAGCGTTAACGAAAAACA<br>GATCTCTCAAGTCCTGAAAGGTCTGCTGGATTCAAAACGCAAT<br>CTGTCGCTGTGA
p190RhoGAP-A pG1 (*Xenopus laevis*)	GGATCCGACCCGAATGTTGACCGTATTAATCTGGTTATCCTGG<br>GCCGTGATGGCCTGGCTCGTGAACTGGCAAATGAAATCCGTGC<br>TCTGTGCACCAACGATGACAAATACGTGATCGATGGTAAAATG<br>TACGAACTGTCACTGCGTCCGATCGAAGGCAACGTCCGCCTGC<br>CGGTGAATAGCTTTCAGACCTCTACGTTCCAACCGCATGGTTG<br>CCTGTGTCTGTATAACTCCAAAGAATCACTGTCGTACGTGGTT<br>GAAAGTATTGAAAAATCCCGTGAAAGCTCTCTGGGCCGCAAAG<br>AAAACCACCTGATTAATCTGCCGCTGACCCTGATCCTGGTTAA<br>TCGTCGCGGTGATACCAGTTCCGAAACGGCACATAGCCTGATT<br>CAGCACGGCCAGCAAGTTGCGTCTAAACTGCAATGTGTCTTTC<br>TGGATGCCGCAAGCACCGGTCTGGGTTATGGTCGTAACATCAA<br>CGAAAAACAGATCTCACTGATCCTGCGTGGTCTGCTGGAATCG<br>AAACGCAACCTGAATCTGTGA
p190RhoGAP-A pG1 (*Danio rerio*)	GGATCCGACGCAAAAGTGGACCGTATCAACCTGGTCATTCTGG<br>GTAAAGATGGCCTGGCTCGTGAACTGGCGAACGAAATCCGTGC<br>CCTGTGTACCAACGATGACCATTATGTCCTGGAAGGTCGTATG<br>TACGAACTGGTTCTGCGCCCGATTGAAGGCAACGTCCGTCTGC<br>CGGTGAACAGCTTTCATACCTCGACGTTCACCCCGCACGGTTG<br>CCTGTGTCTGTATAACAGTAAAGAAAGTCTGTCCTACGTGGTT<br>GAAAGTATTGAAAAACTGCGCGAATCCACGCTGGGCCGTCGCG<br>ATAATCACATGGCACAGCTGCCGCTGAGCCTGCTGCTGGTTTC<br>TAAACGTGGTGTCGGCGGTCTGTCAGACATCGGCGGTGAAACC<br>GCGCAGGCCCTGATTTCGCAAGGCCAGCAAGTGGCAATCAAAC<br>TGCAGTGCAACTATCTGGAACCGAGCTCTCCGGGTACCGGTTA<br>TGGTCGTAACGTGAACGAAAAACAGATCAACCAAGTTCTGAAA<br>GCTCTGCTGGAAACCCGTCGCACGTCTACCTTTTGA
p190RhoGAP pG1 (*Drosophila melanogaster*)	GGATCCGGCTCGGACCGCACGCTGAACCTGCTGATTGTGGGCT<br>CGGAACACCTGGCATCTGACCTGCTGAACGACATTCGCATCTG<br>TACGGGTAGCAAAGGCGAATATATTTACGAAAACCAGACCTAT<br>TACCTGAACTATCGTATCGCGAATGGCGATATGGAAGCGTTTA<br>AAGCCATTGACGTCTATAGCTCTGGTCTGATCTGCGTGTACAG<br>TAACCAGCAATCCTTCGAAACCCTGAAAGATAACCTGGAACGC<br>ACGCTGCTGTGTAATCTGGAACTGGAAGACAAATTTGAAAATC<br>TGCCGATTGTGCTGGTTTATCAGCCGCAAGATCTGAAAGAAAA<br>CGAAGTTGAATACCTGCGTAATGAGGGTATGCGCCTGAGCGAA<br>ATGCTGCATTGCGATTTCATCGACCATACGCAGAATCACCAAA<br>AATACGTCTATGACATCCTGAACATCGTGATTCTGAGCCTGAA<br>ACTGACCGAAATGAAATGA

Supplementary Table 4. Primer Sequences.

pCDNA Flag-p190RhoGAP-A	Forward	5′-GCAGGATCCTATGATGATGGCAAGA-3′
	Reverse	5′-CTGGAATTCTCACAGCGTGTGTTC-3′
pG1 p190RhoGAP-A (residues 592-767)	Forward	5′-GAAGGATCCGACCCCAATATTGAT-3′
	Reverse	5′-CCTGAATTCCTACAGGTTTAAGTTGCG-3′
pG2 p190RhoGAP-A (residues 766-960)	Forward	5′-GAAGGATCCAACCTGGTTAGTTCT-3′
	Reverse	5′-CCTGAATTCCTAAGCCACATTATCGTAC-3′
pG1 p190RhoGAP-B (residues 590-763)	Forward	5′-GAAGGATCCAGTACCAATATAGAT-3′
	Reverse	5′-CCTGAATTCCTACACATCCAAATTGTG-3′
pG2 p190RhoGAP-B (residues 764-954)	Forward	5′-GAAGGATCCGTGAGCCCAATTCCT-3′
	Reverse	5′-CCTGAATTCCTATGTATTATCAGACAA-3′
pCDNA Flag-p190RhoGAP-A ΔpG1-pG2 mutagenesis	Forward	5'-TAGGAATCAGAAGAACTCTTTGTCTTGCAGCACCACTG-3'
	Reverse	5'-CAGTGGTGCTGCAAGACAAAGAGTTCTTCTGATTCCTA-3'

Supplementary References

1. Kabsch, W. & Sander, C. Dictionary of protein secondary structure: pattern recognition of hydrogen-bonded and geometrical features. *Biopolymers* **22**, 2577-637 (1983).
2. Bond, C.S. & Schuttelkopf, A.W. ALINE: a WYSIWYG protein-sequence alignment editor for publication-quality alignments. *Acta crystallographica. Section D, Biological crystallography* **65**, 510-2 (2009).
3. Landau, M. et al. ConSurf 2005: the projection of evolutionary conservation scores of residues on protein structures. *Nucleic Acids Res* **33**, W299-302 (2005).
